# Current techniques for severe mitral annular calcification

**DOI:** 10.1016/j.xjtc.2023.10.004

**Published:** 2023-10-07

**Authors:** Jessica K. Millar, Gorav Ailawadi

**Affiliations:** a Department of Surgery, University of Michigan, Ann Arbor, Mich; b Department of Cardiac Surgery, University of Michigan, Ann Arbor, Mich

**Keywords:** mitral annular calcification, transcatheter mitral valve replacement, transcatheter edge-to-edge repair

**Figure:**
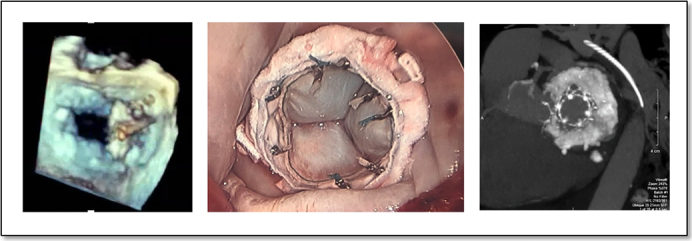
New techniques and technology for the treatment of severe mitral annular calcification.

**Central Message**

Ongoing development of surgical and transcatheter approaches have helped advance treatment of mitral valve disease in the setting of mitral annular calcification (MAC).

Mitral annular calcification (MAC) remains a technically challenging disease process, with increased morbidity and mortality in the setting of mitral valve replacement (MVR) compared with mitral valve surgery without MAC.^1^^,^^2^ MAC can vary significantly in pattern and severity, creating diagnostic and technical challenges. It may be classified based on segment extension, with single-segment extension affecting P2 being the most prevalent.^3^ Alternatively, it may be classified by circumferential spread, comprising type 1 (partial MAC; <270° annular calcium), type 2A (≥270° annular calcium with absence of predicted left ventricular outflow tract [LVOT]), and type 2B (≥270° annular calcium with presence of predicted LVOT).^4^ However, there is no universally accepted approach to surgically address MAC.^5^ As such, various approaches have been employed to address MAC, including complete resection of all calcifications with annular reconstruction, partial resection with removal of enough calcium to adequately seat a bioprosthetic valve, and transcatheter valve-in-MAC approaches.^6^ However, as recognition and referral for MAC disease continues to grow, re-evaluation and incorporation of new techniques and technology to address MAC are necessary. Here, we present current strategies to address severe MAC through several case examples. Images were obtained following signed informed consent from each patient. All noncontributory personal health information and patient identifiers were removed.

## Case Presentations and Surgical Techniques

### Ultrasonic Emulsification

**Figure 1:**
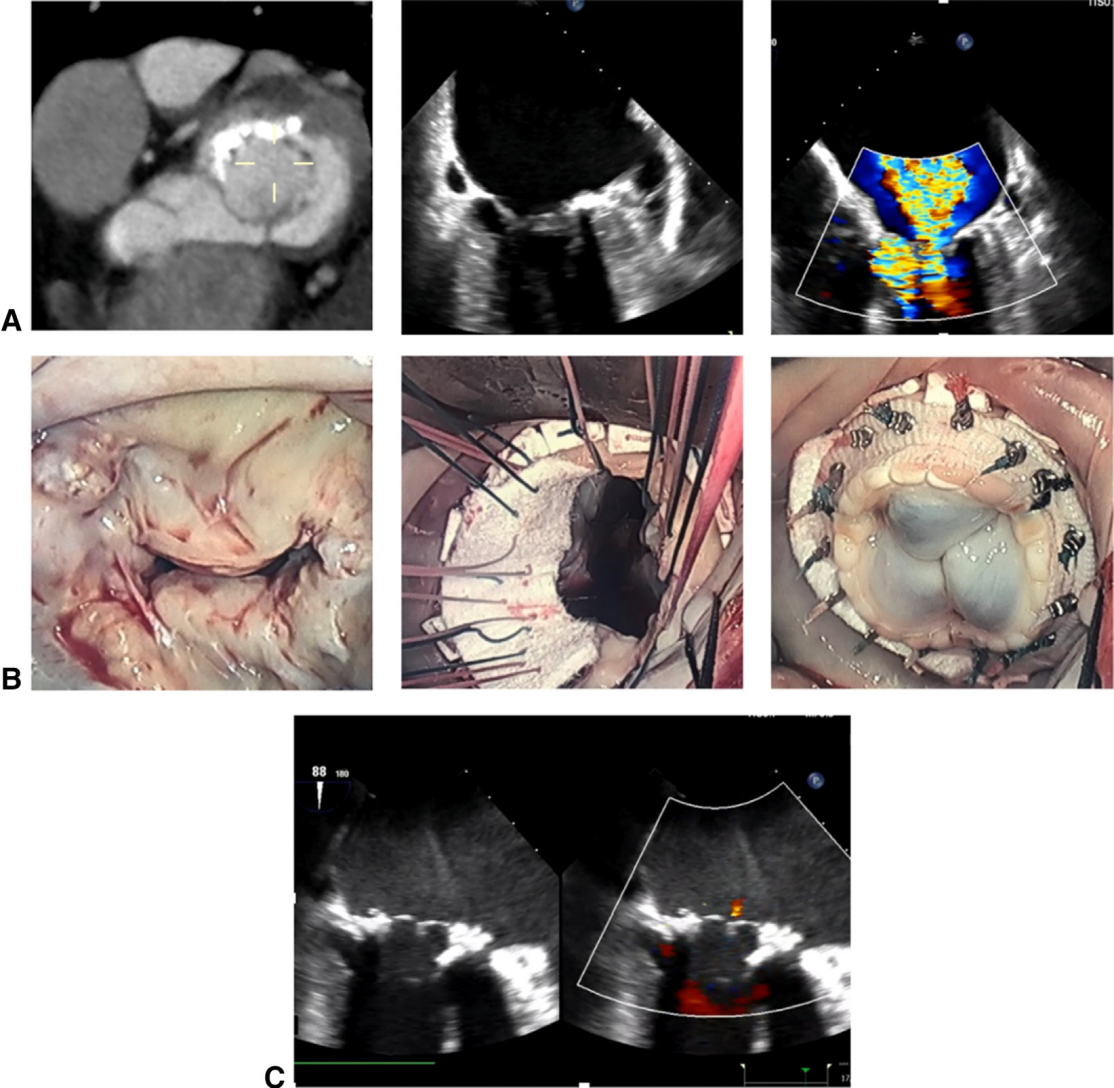
Use of ultrasonic emulsification. A, Preoperative CT and 2D echocardiogram demonstrating focal MAC (*right* and *middle panel*) with MR (*left panel*). B, Intraoperative images demonstrating initial MAC (*right panel*), followed by placement of a 29-mm Epic valve via a minimally invasive approach (*middle* and *left panel*). C, Postoperative 2D echocardiogram demonstrating resolution of MR.

A 71-year-old female patient with a history of rheumatic mitral regurgitation (MR) and mitral stenosis (MS), focal MAC (Figure 1, *A*), and New York Heart Association Class 3 heart failure underwent a minimally invasive MVR. During the procedure, debridement of focal calcium was performed using ultrasonic emulsification via a Sonopet ultrasonic aspirator (Stryker) before placement of a 29-mm Epic valve (Abbott Laboratories) (Figure 1, *B* and *C*). She recovered well without heart failure for several years postoperatively.

Ultrasonic emulsification/aspiration provides a method of controlled, purposeful debridement and allows for “remodeling” of the annulus to an extent sufficient enough to seat an adequately sized prothesis.^6^ With such a technique, complete resection and excessive manipulation of MAC is not necessary. In addition, it allows for the avoidance of excessive torque on the annulus, thus minimizing the risk of atrioventricular groove disruption. Placement of sutures with the severely calcified annulus can be challenging. Removal or softening of the calcium can permit easier suture placement, and unconventional suture placement (such as placement in the left atrial wall) may be necessary to ensure equal distribution of forces along sutures. In our previously reported experience, ultrasonic emulsification/aspiration allows for safe treatment of patients with severe MAC using conventional, suture-based mitral valve prosthesis.^6^

### Transcatheter Edge-to-Edge Repair

**Figure 2:**
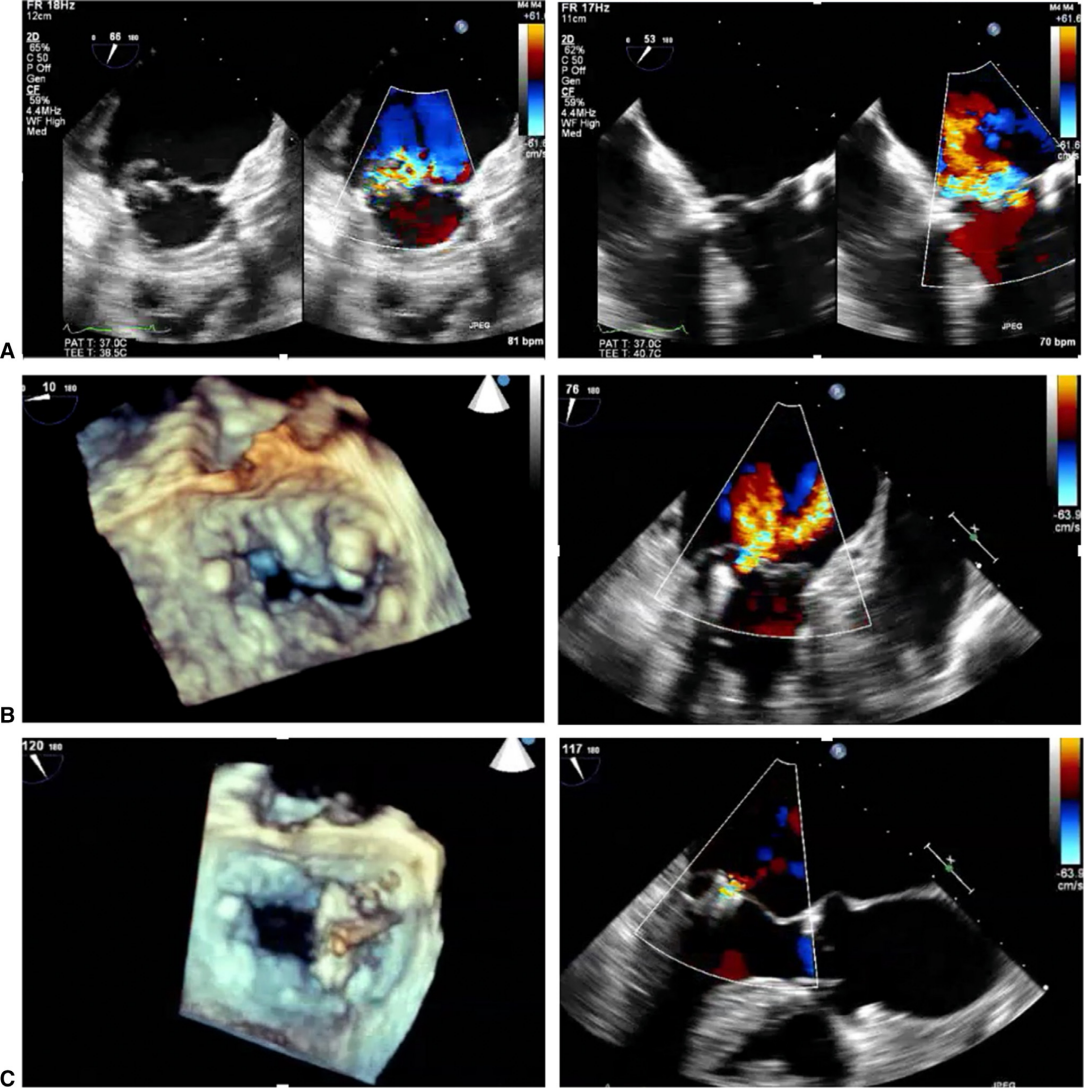
Transcatheter edge-to-edge repair with native MAC. A, Preoperative echocardiogram demonstrating severe MR, flail P3 segment, and moderate focal posterior MAC. B, 3D (*right panel*) and 2D echocardiogram (*left panel*) demonstrating placement of first MitraClip. C, 3D (*right panel*) and 2D echocardiogram (*left panel*) demonstrating placement of second MitraClip.

An 82-year-old frail female patient with a history of sarcoidosis presented with severe MR. On preoperative evaluation, she was found to have an ejection fraction of 70%, an elevated pulmonary artery pressure of 96 mm Hg, and a flail P3 segment resulting in her severe MR (4+). In addition, she was found to have a focal area of moderate MAC and a valve area of 4.5 cm^2^ (Figure 2, *A*). Given her overall health status, she underwent transcatheter edge-to-edge repair (TEER) with placement of 2 MitraClip devices (Abbott Laboratories) in the commissure with trace residual MR (Figure 2, *B* and *C*).

Due to the high surgical morbidity and mortality associated with MAC, less-invasive yet efficient alternatives to surgery are needed. TEER with MitraClip placement in select patients has been demonstrated to be a safe and feasible alternative to achieve significant reduction of MR and substantial clinical improvement in high-risk surgical patients.^7^ Recent retrospective studies demonstrated similar procedural success (91.8% in MAC vs 95.1% in non-MAC) and incidence of procedural complications, no difference in 1-year cardiovascular mortality (15.3% in MAC vs 9.2% in non-MAC), and ability to achieve an MR grade ≤2 at 1-year follow-up (90.6% in MAC vs 79.5% in non-MAC) following TEER.^7^

Challenges to TEER in the setting of MAC include retraction of the posterior leaflet, extension of calcium onto the leaflets, or small native valve area. MAC may cause such significant posterior leaflet retraction that less than 5 mm of tissue is available to grasp and secure attachment may not be possible.^8^ Similarly, extension of calcium onto the leaflet may increase the risk of leaflet tear.^8^ In the case presented, that patient's MAC was focal with an adequate leaflet grasping zone free of calcium. In such cases with acceptable anatomy, TEER may be considered as a valid alternative to surgery in select, high-risk patients with severe MR and MAC.

### Surgical Implantation of Transcatheter Aortic Valve

**Figure 3:**
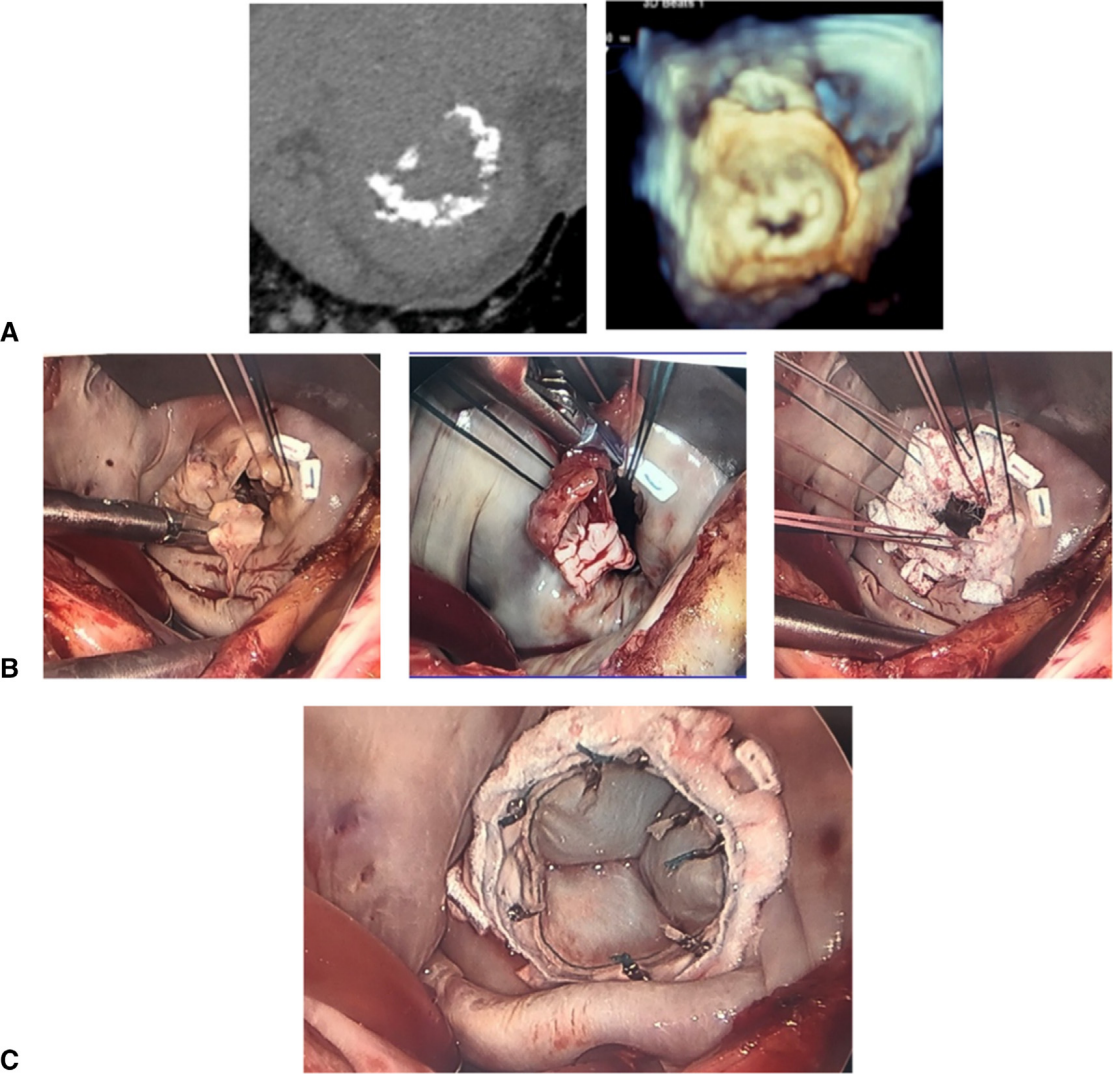
Surgical implantation of a transcatheter valve. A, Preoperative CT and 3D echocardiogram demonstrating severe MAC. B, Intraoperative images demonstrating removal of A2 segment (*right panel*), septal resection (*middle panel*), and placement of felt strip (*left panel*). C, Final implantation of Sapien 3 valve.

An 81-year-old female patient with a history of previous 4-vessel coronary artery bypass grafting and aortic valve replacement (2011) presented years later with severe MS (mean gradient 14 mm Hg) in the setting of severe MAC (Figure 3, *A*) and elevated pulmonary artery pressures (68 mm Hg). She underwent a minimally invasive MVR with placement of a Sapien 3 valve (Edwards Lifesciences) (Video 1). During this procedure, the A2 segment and papillary muscle were resected and a septal resection was performed to minimize LVOT obstruction (Figure 3, *B*).^9^ Pledgeted annular sutures were placed through the MAC with 2 redundant felt strips anchored circumferentially in the landing zone to help minimize paravalvular leak (Figure 3, *B*). The valve was placed 60% to 70% within the left atrium to avoid LVOT obstruction, and the annular sutures were secured to the frame of the valve (Figure 3, *C*). The patient recovered well and remained without hospitalization for 4 years.

Transcatheter mitral valve replacement (TMVR) with balloon-expandable valves can be a suitable approach for select patients with severe MAC. Previous multicenter global registry studies evaluating outcomes following TMVR with balloon-expandable valves (BEVs) in patients with severe MAC demonstrated a high rate of technical success (72%) but a concurrent high rate of adverse events (29.7%).^10^ The use of balloon-expandable aortic valves in MAC has the advantage of minimizing suture placement and complications of debridement but can result in significant paravalvular leak and LVOT obstruction and, therefore, remains an alternative for selected high-risk patients with limited treatment options.^10^ These risks are greatest when performed via a transeptal percutaneous approach; however, surgical transatrial TMVR can minimize LVOT obstruction and paravalvular leak using the techniques described here. Early results have demonstrated a technical success rate of 94.4%, with paravalvular leak and LVOT obstruction (especially in cases without septal myomectomy) comprising the greatest risks.^11^ Importantly, these results also demonstrated a greater risk of mortality in patients with significant MR versus isolated MS (hazard ratio, 2.32).^11^ As such, those patients with MR associated with MAC may benefit less from TMVR than patients with isolated MS.

The Surgical Implantation of Transcatheter Valve in Native Mitral Annular Calcification (SITRAL) study (ClinicalTrials.gov Identifier: NCT02830204) is an ongoing study to evaluate the safety and feasibility of surgical implantation of BEVs in patients with MAC who are at high risk for mitral valve surgery or deemed inoperable due to the extent of calcification.^12^ This hybrid approach offers a unique treatment option for those patients with mitral valve disease complicated by severe MAC who may be at increased risk for other conventional approaches or at risk for LVOT obstruction with TMVR. This open surgical implantation approach has been demonstrated to have a 30-day mortality rate of 13.7% and a technical success rate of 94.1%.^13^ However, there remains anatomical limitations to this approach, as patients with large native annulus are at increased risk for paravalvular leak.^13^

### Tendyne TMVR

**Figure 4:**
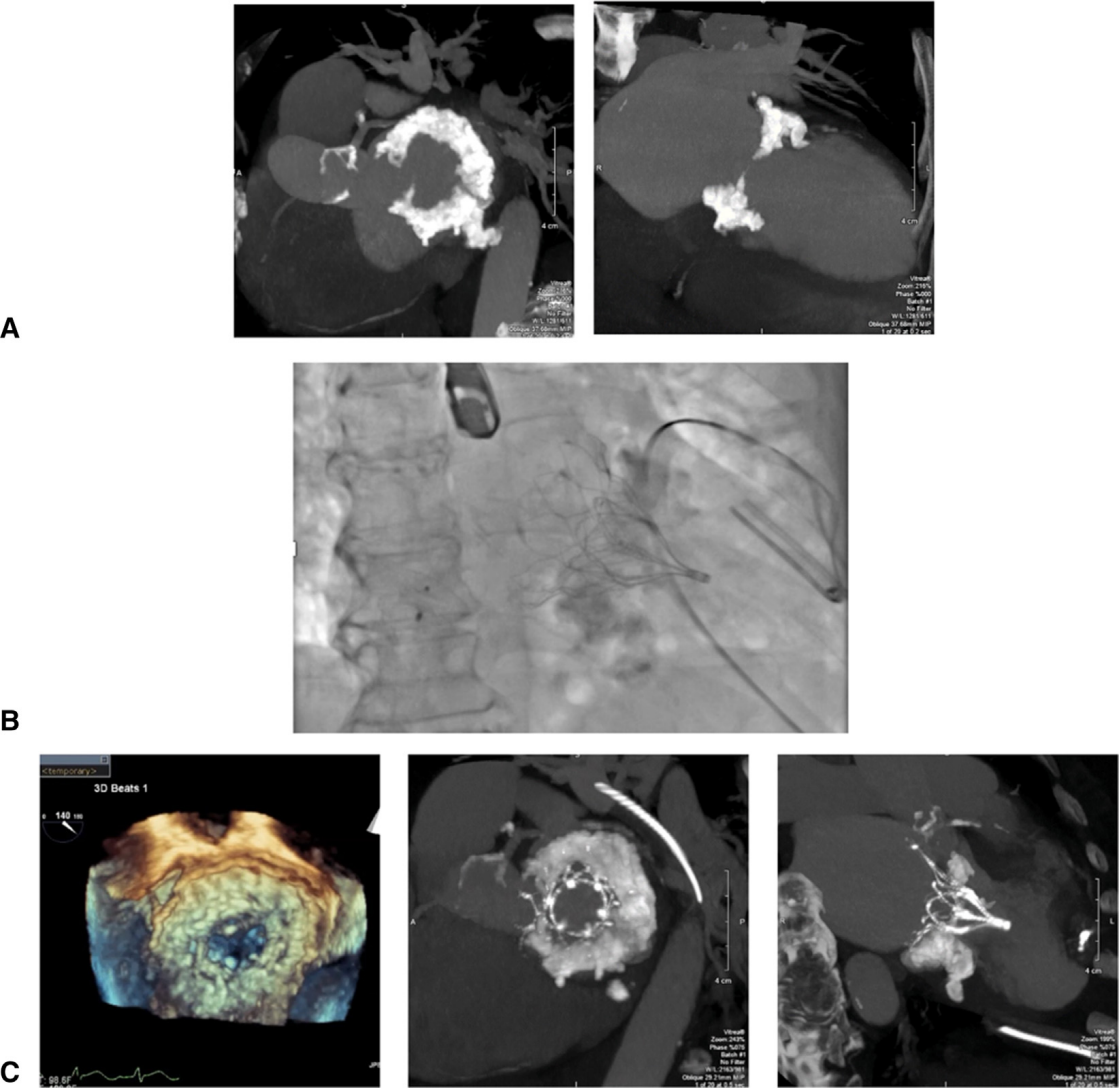
Tendyne MVR A, Preoperative CT demonstrating severe MAC. B, Intraoperative fluoroscopy demonstrating placement of Tendyne valve. C, Postoperative 3D echocardiogram and CT demonstrating placement of Tendyne valve.

A 75-year-old female patient with no medical history presented for acute congestive heart failure secondary to MR in the setting of severe MAC (Figure 4, *A*). Given the degree of MAC, she was not a candidate for any option described thus far. She underwent transapical placement of a 34-Fr Tendyne valve (Figure 4, *B* and *C*). This approach uses an investigational device contoured to fit the mitral valve annulus. The valve is composed of an outer and inner frame as well as an anchoring tether/hemostatic pad, allowing it to be placed transapically. Uniquely, the valve is both retrievable and repositionable. This device is currently limited to investigational use only as part of the Clinical Trial to Evaluate the Safety and Effectiveness of Using the Tendyne Transcatheter Mitral Valve System for the Treatment of Symptomatic Mitral Regurgitation (SUMMIT; ClinicalTrials.gov Identifier: NCT03433274).^14^ Patients with moderate-to-severe MR as well as those with symptomatic mitral valve disease due to severe MAC who would otherwise be deemed unfit for mitral valve surgery are eligible and may offer to new insight to emerging TMVR devices.

## Conclusions

Several current and emerging strategies exist for patients requiring mitral valve intervention with MAC. Although these patients present with unique technical challenges and carry with them elevated operative risks, meaningful clinical improvements are possible, and appropriate intervention should still be pursued. Ultrasonic emulsification/aspiration can be used to focally debride calcium and can make conventional repair/replacement feasible. In high-risk patients with a valve area >4 cm^2^, TEER may still be feasible with focal MAC and adequate amount of unaffected leaflet tissue. In patients with MS and MAC, surgical BEVs may the optimal approach. In patients with MR and MAC, ongoing TMVR trials with mitral-specific devices may provide novel treatment options. Institutional capabilities and anatomic exclusions may limit these options for some patients, and some may still require traditional surgical approaches. However, the ongoing development of surgical and transcatheter approaches has increased the toolbox for tailored treatment strategies and improved patient care.

## Conflict of Interest Statement

The authors reported no conflicts of interest.

The *Journal* policy requires editors and reviewers to disclose conflicts of interest and to decline handling or reviewing manuscripts for which they may have a conflict of interest. The editors and reviewers of this article have no conflicts of interest.

## Supplementary Data

Video 1 Surgical implantation of transcatheter valve. Discussion of the technique details, tips, and troubleshooting strategies during surgical implantation of a transcatheter valve in native MAC. Video available at: https://www.jtcvs.org/article/S2666-2507(23)00382-6/fulltext.
