# Supplementary figures and images for: Current techniques for severe mitral annular calcification
Source: JTCVS Tech. 2023 Oct 7;22:53–8. doi: 10.1016/j.xjtc.2023.10.004 (PMC10750962; doi:10.1016/j.xjtc.2023.10.004)

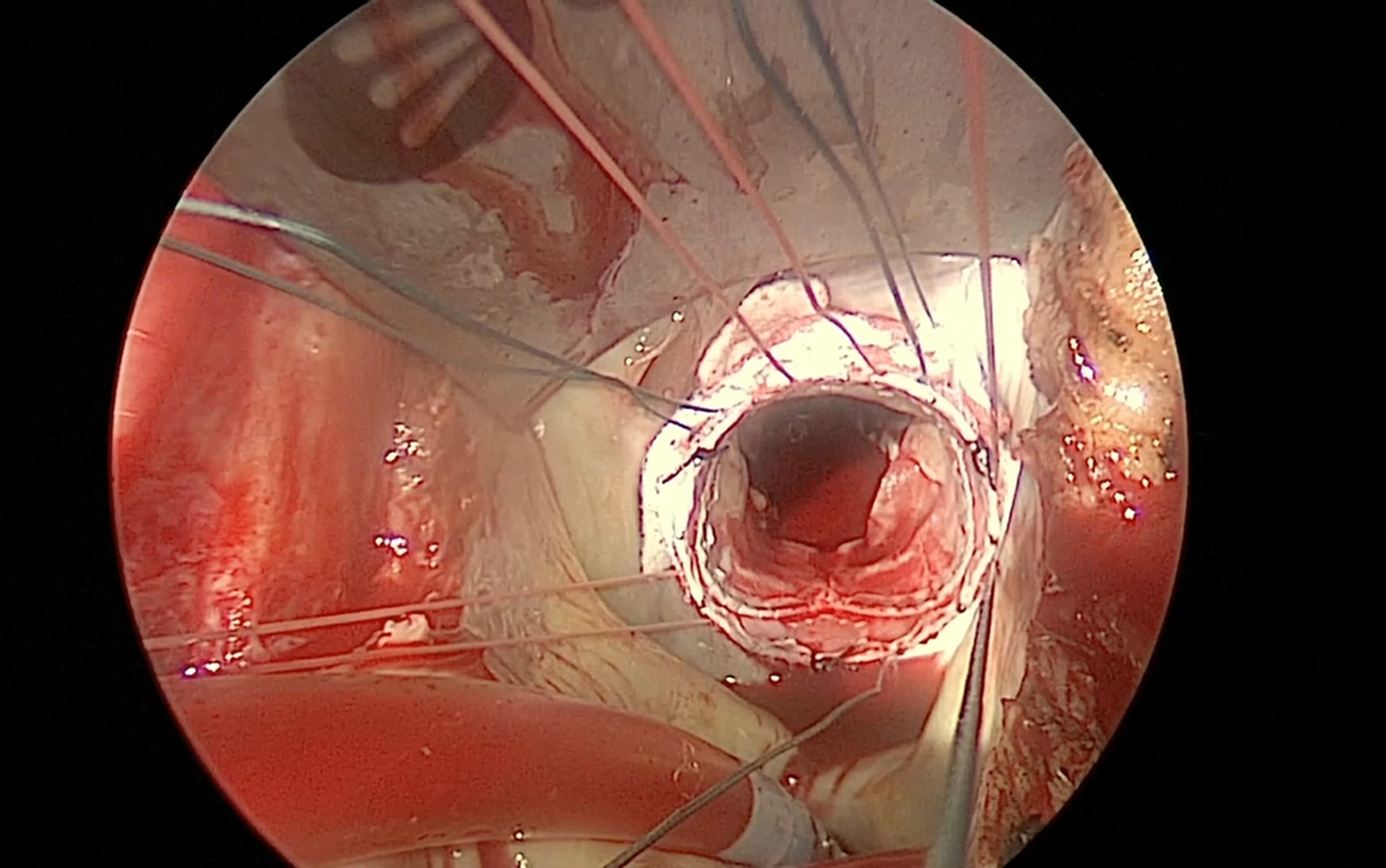

Supplement: Video 1 — Surgical implantation of transcatheter valve. Discussion of the technique details, tips, and troubleshooting strategies during surgical implantation of a transcatheter valve in native MAC. Video available at: https://www.jtcvs.org/article/S2666-2507(23)00382-6/fulltext. [file fx2.jpg]
